# An International Systematic Review of Prevalence, Risk, and Protective Factors Associated with Young People’s E-Cigarette Use

**DOI:** 10.3390/ijerph191811570

**Published:** 2022-09-14

**Authors:** Jinyung Kim, Serim Lee, JongSerl Chun

**Affiliations:** Department of Social Welfare, Ewha Womans University, 52, Ewhayeodae-gil, Seodaemun-gu, Seoul 03760, Korea

**Keywords:** adolescent, e-cigarette, prevalence, global health, protective factors, risk factors

## Abstract

While the prevalence of young people’s conventional cigarette use has decreased in many countries, the use of e-cigarettes has risen. To effectively counteract the growing popularity of e-cigarettes among young people internationally, researchers should know the exact prevalence as well as the protective and risk factors associated with vaping. Based on five eligibility criteria, 53 articles were chosen and analyzed by general characteristics, prevalence, sample characteristics, gender difference, protective factors, and risk factors. In this study, the international pooled prevalence of young people’s lifetime e-cigarette use was 15.3%, the current use was 7.7%, and dual use was 4.0%. While the highest lifetime, current, and dual prevalence were found in Sweden, Canada, and the United Kingdom, respectively, the lowest prevalence was found in Germany, followed by South Korea and Sweden. Some protective and risk factors include perceived cost and danger of vaping, parental monitoring, internal developmental assets, cigarette use, family and peer smoking, exposure to online advertisements, and the presence of nearby retail stores. Based on this review, researchers and practitioners can develop different intervention programs and strategies for young smokers.

## 1. Introduction

The use of electronic cigarettes (e-cigarettes) among young people, including adolescents and emerging adults, is a rising concern worldwide. E-cigarettes are handheld, small, rechargeable, and battery-powered electronic devices that deliver nicotine to users through aerosols or vapors [1,2]. Since their invention in the early 2000s, e-cigarettes have become trendy among young people [1,3]. Fadus et al. [3] indicate a swiftly increasing trend in e-cigarette use, while traditional combustible cigarette use has decreased slightly. In addition, many studies suggest that the use of e-cigarettes is an international phenomenon and is increasing globally [4,5]. For instance, the past 30-day use of e-cigarettes among young Americans rose from 1.5% in 2008 to 20.8% in 2018 [6]. To be more specific, the highest ever e-cigarette use was reported among young adults (aged 20–28), followed by adults (above 18) and adolescents (aged 11–19), in the United States [7]. In Asia, the rate of young Korean who had ever used e-cigarettes rose from 0.5% in 2008 to 42% in 2017 [8,9]. Similarly, the current e-cigarette use of young Taiwanese men grew from 2.5% in 2014 to 6.4% in 2017 [10]. In European countries, the prevalence of having ever used e-cigarettes varied by 17–62% [1,5,11]. Vapers in many European countries were previously cigarette users or started smoking at a young age, and some of these nations even reported a higher prevalence of e-cigarette use than traditional cigarette use (e.g., Iceland, Monaco, Lithuania, Poland, Ireland, Germany, Czechia, Hungary, France, and Norway) [12]. After the implementation of the EU Tobacco Products Directive regulations, e-cigarette use among young people in European nations, including the UK, is slowing down [13,14]. However, unlike other countries, England still shows an increase in past and current e-cigarette use from 2014 to 2016 [13,14]. Despite the significance of these findings, there has been a lack of data on the pooled prevalence of young people’s e-cigarette use. Some studies have attempted to estimate the global prevalence of e-cigarette use; however, they either covered only current use or excluded dual use in their analyses [15,16,17].

Adolescents have been easily fascinated by modern e-cigarettes for the following reasons: (1) sleek designs, (2) user-friendly functions, (3) less aversive smoking experiences, (4) desirable flavors, and (5) the ability to be used discreetly in places where smoking is forbidden [18]. It is believed that e-cigarettes are a safer alternative to smoking [18]. But, growing evidence suggests that e-cigarettes also have physical and developmental harms and may lead to nicotine dependence, just as with traditional cigarette use [19]. For instance, nicotine in e-cigarettes could disrupt the brain development or cardiovascular and respiratory systems of young people, including adolescents and young adults [20]. Further, e-cigarette users are likely to be diagnosed with pneumonia-related diseases, ulcerative colitis, acute myocardial infarction, short-term lung obstruction, and multiple allergic symptoms in children [21,22]. However, the long-term effects of e-cigarettes are still unknown, and more is to be revealed in the upcoming studies [23]. Young people are particularly vulnerable to the harmful impacts of e-cigarettes, and e-cigarette use can lead to conventional cigarette smoking during early adulthood [1]. As for dual users, they report a lower general health score, and more difficulty in breathing in the past month, compared to cigarette-only users [24]. Additionally, a significant difference was observed in the history of arrhythmia between cigarette-only users (14.2%) and dual users (17.8%) [24]. More respiratory symptoms were found in dual smokers than in smokers who did not use e-cigarettes [22], and the possibility of reversible cerebral vasoconstriction syndrome was more likely to be observed in dual users [25].

With a growing public interest in e-cigarettes, many studies have explored this topic, including literature on the protective and risk factors of e-cigarettes [26]. For example, the main predictors of young people’ e-cigarette use include demographic factors (e.g., age, sex, and ethnicity), psychological and personality factors (e.g., internalizing problems, externalizing problems, breaking rules, enjoying frightening things, and preferring unpredictable friends), parental factors (e.g., parental education, family structure, and secondhand smoke exposure at home), and other substance abuse (e.g., alcohol and marijuana) [1]. In addition to these factors, Bowe et al. [27] also found that regular participation in team sports and a higher value placed on conventional social norms significantly decreased the use of both conventional cigarettes and e-cigarettes (or dual use) among young people in Ireland. At the school level, ethnicity in school, the private status of the school, the urbanicity of the school, the and US census region of the school, were significant predictors of e-cigarette use among young people [28]. Researchers also suggest gender differences in e-cigarette use among young people [28,29,30]. However, to date, no study has estimated the international prevalence of young people’s e-cigarette use and systematically reviewed the findings on the protective and risk factors of vaping. Thus, this study calculated the worldwide e-cigarette prevalence and organized a list of protective and risk factors associated with young people’s e-cigarette use.

## 2. Methods

### 2.1. Study Identification

We searched scholarly articles and dissertations published from January 2003 to February 2021 in the most used international academic search engines: Google Scholar, PubMed, EBSCO, and ProQuest [31,32,33]. First, we set the timeframe starting in 2003 to coincide with the first developed e-cigarette device, which was available on the US market in the mid-2000s [34]. To select the relevant manuscripts, we used the following keywords: protective factor, risk factor, predictors, adolescent, youth, student, young people, e-cigarette, vaping, electronic cigarette, e-cigs, e-hookahs, mods, vape pens, vapes, tank systems, and electronic nicotine delivery systems (ENDS). In addition, we used all the e-cigarette-related terms suggested by the Centers for Disease Control and Prevention [35].

### 2.2. Selection Procedure

Following the PRISMA guideline [36], we first identified 206,867 records through database searching and then removed duplicates. Of the 159,014 records, we removed 130,697 because the title and keywords did not match the study’s aim. By screening the abstracts, we excluded 27,072 records. Out of 1245 full-text articles assessed for eligibility, we excluded 1198 because they did not meet the eligibility criteria. There were four eligibility criteria in this review: (1) examining either protective or risk factors of e-cigarette use, (2) measuring lifetime, current, or dual e-cigarette use as the outcome variable, (3) published in English, and (4) targeting young people (9–25 years old) [37]. All records had to be peer-reviewed journal articles and doctoral dissertations. 

Researchers conducted a quality assessment for the selected studies to ensure their “relevance, methodological rigor, and credibility” [38,39]. Each study was evaluated based on the following questions: (1) “Is the research question clear and adequately substantiated?” (2) “Is the design appropriate for the research question?” (3) “Was the sampling method appropriate for the research question and design?” (4) “Were data collected and managed systematically?” and (5) “Were the data analyzed appropriately?” [38] Each study was scored from one to five points, and only studies with more than four points were included in the analysis. 

**Figure 1:**
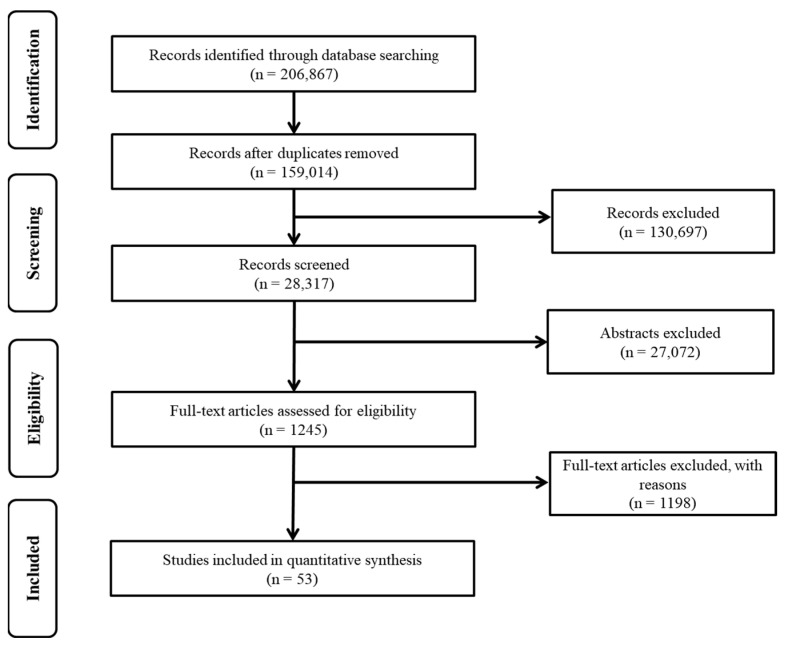
Study selection procedure.

Moreover, to establish the same criteria and time frame for a lifetime, current, and dual e-cigarette use, we have defined lifetime use as “having ever used e-cigarettes, even just once,” current use as “the use of a tobacco product on at least one day in the past 30 days,” and dual use as “the use of both products (i.e., cigarette and e-cigarette) within the same reporting period (e.g., the past 30 days)” [40]. After these stages, a total of 53 articles were chosen for analysis (Figure 1) [1,4,5,8,9,11,23,28,29,30,40,41,42,43,44,45,46,47,48,49,50,51,52,53,54,55,56,57,58,59,60,61,62,63,64,65,66,67,68,69,70,71,72,73,74,75,76,77,78,79,80].

### 2.3. Coding and Analysis

Two raters individually reviewed each article by general characteristics (i.e., year published, study site (country), sampling method), prevalence, sample characteristics, gender differences, protective factors, and risk factors. The coding scheme referred to several other systematic reviews [81,82,83,84]. We organized the list of identified protective and risk factors into individual, family, peer, school, community, and societal levels, following the socio-ecological model (SEM) [85]. We further divided the individual level into three subsections, including status (e.g., academic competence), behavior (e.g., cigarette use), and perception (e.g., perception of the harms of e-cigarettes). If any disagreement arose during coding, the authors reviewed the matrix and discussed the issue to reach an agreement. The Kappa coefficient for the two raters was calculated as the interrater reliability; it was 0.846, indicating a high consistency. Regarding the pooled prevalence, all the prevalence and sample sizes reported in each study were entered into the Comprehensive Meta-Analysis (CMA) software. Because prevalence usually varies by sample size, we assumed that observed estimates would be different across studies; thus, the random-effects model was considered when estimating the pooled prevalence [86].

## 3. Results

### 3.1. General Characteristics

The published studies included in our review were from 2014 to 2021. Out of the 53 studies, 13 (24.5%) were published in 2020, eight (15.1%) in 2016, seven (13.2%) in 2017 and 2019, six (11.3%) in 2015, and three (5.7%) in 2021. Approximately 73% of the selected studies used random sampling (*n* = 39), and 26.4% employed non-random sampling (*n* = 14), but a majority used random sampling methods to select participants. Researchers conducted the studies in 20 different countries. More than 50% of the studies (*n* = 30) were from the United States, and 7.5% were from South Korea (*n* = 4); Canada, Poland, and Sweden each represented 3.8% of the studies (each with *n* = 2). Only six studies (11.3%) were conducted in Asian countries, including South Korea (*n* = 4), Malaysia (*n* = 1), and Thailand (*n* = 1) (Table 1).

### 3.2. Prevalence

**Figure 2:**
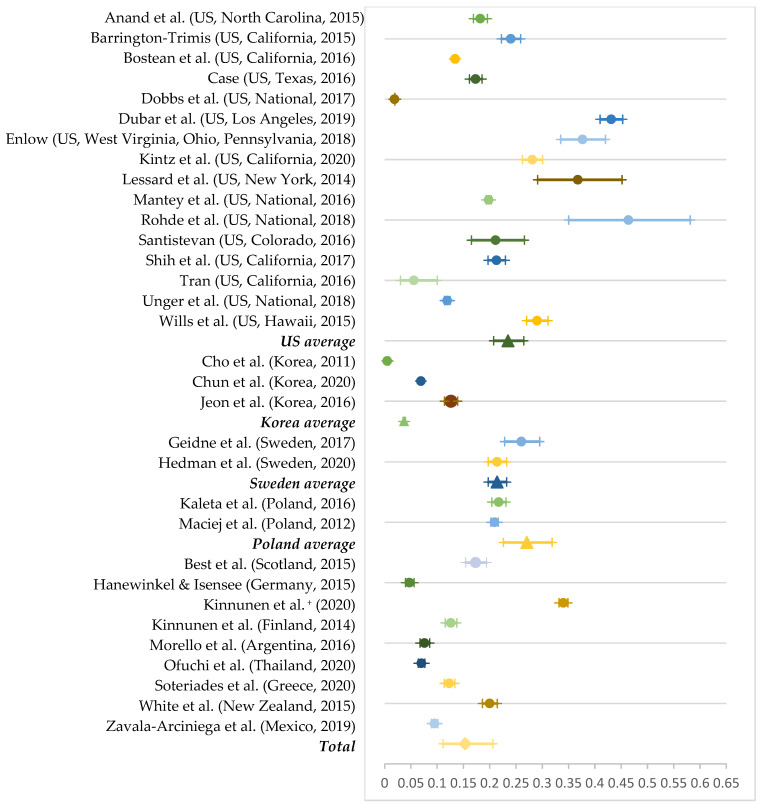
Prevalence of lifetime e-cigarette use by country [4,5,11,19,40,41,42,43,46,47,48,50,51,52,54,56,57,58,59,60,61,62,64,65,67,69,70,71,73,76,79,80]. Note 1. ^+^ Includes six European countries (Belgium, Finland, Germany, Ireland, Italy, Netherlands, Portugal). Note 2. National average is presented with the shape of a triangle and the total pooled prevalence as rhombus.

Except for six studies, all reported the prevalence of either lifetime or current e-cigarette use and current dual use. By country, the average lifetime e-cigarette use was 23.5% in the United States (out of 16 studies), 6.7% in South Korea (out of three studies), 23.7% in Sweden (out of two studies), and 21.3% in Poland (out of two studies). We estimated all other prevalence based on a single study: 34% in European countries, 20% in New Zealand, 17.3% in Scotland, 12.6% in Finland, 12.3% in Greece, 9.5% in Mexico, 7.6% in Argentina, 7% in Thailand, and 4.7% in Germany. We found the highest lifetime e-cigarette use in Sweden and the lowest in Germany. The international average of lifetime e-cigarette use under the random effect model was 15.3% (95% CI: 11.1–20.6%; Figure 2).

**Figure 3:**
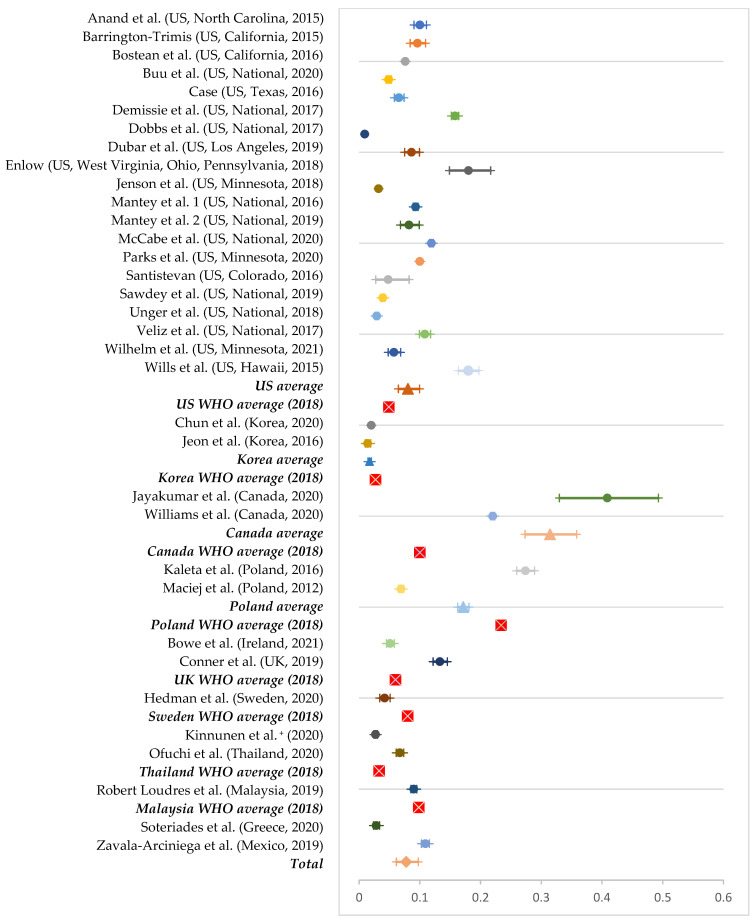
Prevalence of current e-cigarette use by country [11,23,27,28,29,30,40,41,43,44,46,48,49,50,51,52,54,55,56,57,59,62,63,65,66,67,68,70,73,74,77,78,79,80]. Note 1. ^+^ Includes 6 European countries (Belgium, Finland, Germany, Ireland, Italy, The Netherlands, Portugal). Note 2. National average is presented with the shape of a triangle, WHO average as a red box marked with ‘X’, and the total pooled prevalence as rhombus.

For current e-cigarette use, the average rate was 8.04% in the United States (out of 20 studies), 31.4% in Canada (out of two studies), 17.1% in Poland (out of two studies), and 1.7% in South Korea (out of two studies). We based all other estimates on a single study: 13.3% in the United Kingdom, 10.9% in Mexico, 9% in Malaysia, 6.7% in Thailand, 5.1% in Ireland, 4.2% in Sweden, 2.7% in European countries, and 2.8% in Greece. We found the highest current e-cigarette use in Canada and the lowest in South Korea. Compared with the WHO average (i.e., the WHO’s estimated prevalence of e-cigarette use in each country), the rates of current e-cigarette use in this study were higher in the following countries: the United States (8.03% vs. 4.9%), Thailand (6.7% vs. 3.3%), the United Kingdom (13.3% vs. 6%), and Canada (31.4% vs. 10%). The rates of e-cigarette use were lower than the WHO average in Malaysia (9% vs. 9.8%), Poland (17.1% vs. 23.4%), South Korea (1.7% vs. 2.7%), and Sweden (4.2% vs. 8%). Based on these average rates, the international pooled prevalence for current e-cigarette use in this study was 7.7% (95% CI: 6.1-9.7%; Figure 3).

**Figure 4:**
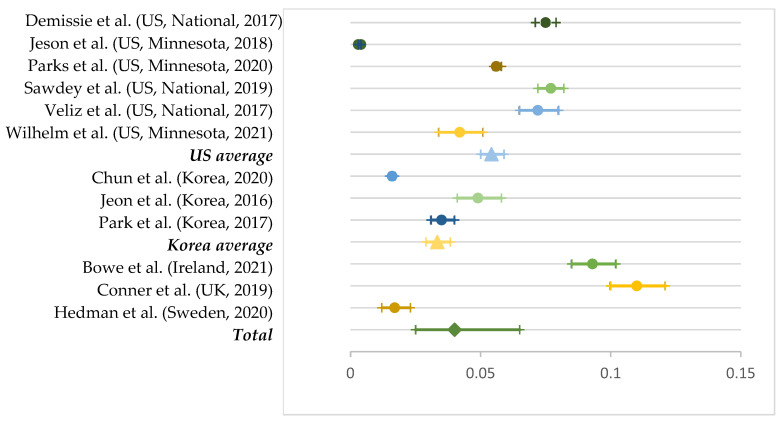
Prevalence of current dual use by country [8,23,27,30,48,49,54,56,66,68,74,77]. Note. National average is presented with the shape of a triangle and the total pooled prevalence as rhombus.

Among seven studies that reported young people’s current dual use, the average was 5.4% for the United States (out of six studies) and 3.3% for South Korea (out of three studies). We calculated all other dual-use prevalence based on a single study: 11% for the United Kingdom, 9.3% for Ireland, and 1.7% for Sweden. We found the highest dual use in the United Kingdom and the lowest in Sweden. Based on these numbers, the international average of dual use was 4.0% (95% CI: 2.5–6.5%; Figure 4).

### 3.3. Sample Characteristics

All studies (*N* = 53, 100%) stated the sample size. The median sample size of 47 studies was 3298 [41], with a minimum of 69 [19] and a maximum of 126,868 participants [23]. The mean sample size was 13,160 (*SD* = 25,920.01). The ages of the sample population ranged from 9 to 25 years old. Most participants in this review were adolescents, and only five [39,41,43,51,58] out of 53 studies included both adolescents and young adults over the age of 20 (Table 1). According to the four classifications of adolescents [87], the most frequently included age group in the studies were middle and late adolescents (i.e., ages 14–19; *n* = 23, 43.4%), followed by mid-stage adolescents (i.e., ages 14–16; *n* = 13, 24.5%), late adolescents (i.e., ages 17–19; *n* = 9, 17.0%), early and middle adolescents (i.e., ages 11–16; *n* = 5, 9.4%), all adolescent age groups (i.e., ages 11–19; *n* = 2, 3.8%), and early adolescents (i.e., ages 11–14; *n* = 1, 1.9%). 

### 3.4. Gender Differences

Of the 53 studies, 67.9% (*n* = 36) reported gender differences, and 32.1% (*n* = 17) did not examine the lifetime or current e-cigarette use between males and females. Out of the 36 studies that examined gender differences, 22 (61.1%) reported statistically significant differences between males and females in e-cigarette use, four (11.1%) estimated the prevalence of e-cigarette use by gender but without statistical analysis, and 10 (27.7%) reported no significant difference. The 22 studies, which reported statistically significant differences, all indicated that males were more likely to use e-cigarettes than females or that being male increases the odds of smoking e-cigarettes. Males had a higher prevalence of lifetime e-cigarette use, current e-cigarette use, and dual use (Table 1).

### 3.5. Protective Factors

Twenty-four (45.2%) studies identified protective factors against young people’s e-cigarette use. This review found some of the common protective factors at the individual and family levels. For instance, at the individual level, the perceived cost and danger of vaping (*n* = 7), engaging in physical activities (*n* = 2), internal developmental assets (i.e., positive identity, empowerment, social competency; *n* = 2), and academic achievement (*n* = 1) played a buffering role against e-cigarette use. At the family level, parental monitoring (*n* = 3), a mother’s higher education level (*n* = 2), and parental support (*n* = 2) reduced the likelihood of vaping (Table 1). Although school and community-level protective factors were examined only in a single study, it is worth noting that young people with high college aspirations and positive teacher engagement were less likely to use e-cigarettes [66,77]. Furthermore, those who did not see or know anyone using e-cigarettes or who lived in a place with high neighborhood cohesion were less susceptible to using e-cigarettes [40,69]. Table S1 (Supplementary Material) provides more information on an additional set of protective factors. In the United States, we found the perceived cost and danger of vaping as the common protective factors [1,19,45,52,61]. 

### 3.6. Risk Factors

All the studies (*N* = 53, 100%) reported the risk factors for young people’s e-cigarette use. Many studies indicated risk factors at the individual level, including cigarette use (*n* = 22), alcohol use (*n* = 16), marijuana use (*n* = 12), other substance/drug use (*n* = 9), sensation seeking (*n* = 6), externalizing problems (*n* = 5), internalizing problems (*n* = 4), poor academic performance (*n* = 4), sexual intercourse (*n* = 3), and perceiving e-cigarettes as less harmful (*n* = 3). Next, we noted family-level factors, such as having family members (e.g., parents, siblings, etc.) as smokers (*n* = 17) and having incarcerated family members (*n* = 2). At the peer level, we found friends smoking (*n* = 16) to be a risk factor, and at the school level, risk factors included attending school with a high portion of students participating in free and reduced lunch programs (*n* = 2). Furthermore, the presence of retailers near schools (*n* = 2) was associated with an increase in young people’s vaping. Exposures to online commercials/advertisements on e-cigarettes and/or cigarettes (*n* = 6) also heightened the risk of e-cigarette use (Table 1).

Table S1 (Supplementary Material) lists other significant factors. By country, we found marijuana and alcohol use to be significantly correlated with e-cigarette use for many participants in the US setting [1,28,68,79]. In addition, two studies from South Korea confirmed that sexual intercourse was a significant factor in increasing the risk of vaping among Korean adolescents [8,48], and waterpipe and snus use were significant risk factors reported in northern European countries such as Sweden and Finland [5,58]. 

**Table 1:** List of protective and risk factors associated with adolescents’ e-cigarette use.

Author (Country)	Prevalence	Sample Size (Age)	Gender Difference	Protective Factors (β/OR)	Risk Factors (β/OR)
Anand et al. (2015) [40] (US)	Lifetime, 18.2%; Current, 10%	3298 (9–12th grades)	Significant difference	Being female (0.59), plan to graduate high school (0.17), mother living in the household (0.55), mother never smoked tobacco (0.50), father never smoked tobacco (0.57), not knowing anyone who uses e-cigarettes (0.25)	Father’s use of snuff (3.82), mother’s use of e-cigarettes (2.60), cigarette use (8.79), smokeless tobacco use (3.75)
Barrington-Trimis et al. (2015) [41] (US)	Lifetime, 24%; Current, 9.6%	2084 (11–12th grades)	Different but not statistically significant	N/A	Anyone living at home using e-cigarettes (6.80) and cigarettes (2.79), number of friends who use e-cigarettes (18.7) and cigarettes (7.46), best friends’ positive reactions to e-cigarette use (18.6), perception of the harm of e-cigarettes and cigarettes (6.02)
Best et al. (2015) [42] (Scotland)	Lifetime, 17.3%	1404 (Pupils in secondary 2 and 4)	N/A	Having never smoked (0.10)	Recognizing more cigarette brands (1.20), having a best friend who smoked (3.17)
Bostean et al. (2016) [43] (US)	Lifetime, 13.4%; Current, 7.6%	67,701 (7, 9, 11th grades)	Significant difference	Being female (0.84)	Parents’ education levels (1.44), having ever used tobacco (6.84), having ever used alcohol (5.83), having ever used marijuana (8.15), race (Hispanic; 1.54), presence of a retailer near schools (1.70), attending schools with a high percentage of students eligible for free and reduced lunch program (2.94)
Bowe et al. (2021) [27] (Ireland)	Current, 5.1%; Dual, 9.3%	4490 (15–16 years old)	Significant difference	Parental supervision (0.71), valuing conventional social norms (0.68)	Parent smokes (1.71), feeling the need to smoke to fit in with peers (2.13), few friends who smoke (2.15), most/almost all friends who smoke (5.19), self-reported academic achievement average (1.43) and below average (2.53), parental reaction to cigarette use (do not care) (4.65)
Buu et al. (2020) [44] (US)	Current, 4.86%	9258 (12–17 years old)	Significant difference	Being non-Hispanic and Black (0.38)	Higher levels of internalizing (1.29) and externalizing problems (1.42), receiving more money per week (1.12), being older (1.72)
Carey et al. (2019) [45] (US)	N/A	3907 (6, 8, 10th grades)	N/A	Positive affect (0.61), belief that e-cigarettes are harmful to health (0.69)	E-cigarette use among family members (4.72), alcohol and marijuana use (3.92), poor school performance (12.98), sensation seeking (1.45), social norm (okay to use, common to use) (6.69)
Case (2016) [46] (US)	Lifetime, 17.3%; Current, 6.55%	3769 (6, 8, 10th grades)	No difference	N/A	Sensation seeking (1.32)
Case et al. (2020) [47] (US)	N/A	2272 (14–18 years old)	N/A	N/A	Higher recall ENDS marketing (1.64), peer tobacco use (3.06), alcohol use (2.67), having ever used marijuana with JUUL (10.08) and with other ENDS (12.07)
Cho et al. (2011) [9] (Korea)	Lifetime, 0.5%	4341 (Middle and high school students)	Significant difference	N/A	Propensity to be easily affected by friends (3.9), having ever smoked a cigarette (11.2)
Chun et al. (2020) [48] (Korea)	Lifetime, 6.9%; Current, 2%; Dual, 1.6%	62,276 (13–18 years old)	Significant difference	Being a vocational school student (0.66)	Tobacco accessibility (1.3), secondhand smoke exposure at home (1.09), sexual intercourse (1.25), being a middle school student (2.13)
Conner et al. (2019) [30] (UK)	Current, 13.3%; Dual, 11%	3210 (13–14 years old)	Significant difference	N/A	Higher impulsivity (1.26), friends and family smoking (1.48), being male (1.64)
Demissie et al. (2017) [49] (US)	Current, 15.8%; Dual, 7.5%	15,624 (9–12th grades)	Significant difference	Engaging in daily physical activity (0.91)	Engaging in a physical fight (1.72), lifetime suicide attempt (1.86), texting or emailing while driving (1.39), drinking alcohol (2.62), using marijuana (3.70), using other illicit drugs (2.73), using nonmedical drugs (2.30), having multiple sexual partners (2.35), being sexually active (1.86), drinking more soda (1.35)
Dobbs et al. (2017) [50] (US)	Lifetime, 19.4%; Current, 9.2%	27,294 (9–19 years old)	N/A	N/A	Perceived e-cigarette as less harm (2.40), perceived less addictiveness of e-cigarettes (2.11), smoking history (7.84), living with a smoker (1.44), being older (1.85), being Hispanic (1.33)
Dubar et al. (2019) [51] (US)	Lifetime, 43.11%; Current, 8.62%	2039 (16–20 years old)	N/A	N/A	Cigarette use (0.17), marijuana use (0.03)
Enlow (2018) [52] (US)	Lifetime, 37.7%; Current, 18%	494 (13–18 years old)	N/A	Perceived costs of vaping (0.52), greater self-efficacy (0.22)	Cigarette use (2.86), alcohol use (2.67), marijuana use (2.23), more modeling of smoking in their social network (1.34), higher extraversion (2.20)
Etim et al. (2020) [53] (US)	N/A	1060 (15–20 years old)	N/A	N/A	Peer e-cigarette use (2.01), exposure to e-cigarette commercials (1.27), household smoking (4.70)
Geidne et al. (2017) [5] (Sweden)	Lifetime, 26%	665 (15–16 years old)	No difference	N/A	Smoking conventional cigarettes (5.6), snus use (2.2), alcohol use (4.4), water pipe use (3.2)
Hanewinkel & Isensee (2015) [4] (Germany)	Lifetime, 4.7%	2693 (5–10th grades)	No difference	N/A	Higher sensation seeking (2.24), having friends (2.06) and parents (1.89) who smoke cigarettes
Hedman et al. (2020) [54] (Sweden)	Lifetime, 21.4%; Current, 4.2%; Dual, 1.7%	2185 (14–15 years old, 19 years old)	Significant difference	Eating a healthy diet (0.74)	Daily smoking (6.27), participation in an arts vocational program (2.22)
Jayakumar et al. (2020) [55] (Canada)	Current, 41%	137 (16–25 years old)	No difference	Perceiving moderate or great risk of regularly vaping without nicotine (0.34)	Current alcohol use (2.66), current cannabis use (13.78), having friends who used cannabis (3.80), using e-cigarettes (2.34), having friends who smoke (2.23), seeing anyone use an e-cigarette in the past seven days (5.97), currently not using cannabis (3.80)
Jeon et al. (2016) [56] (Korea)	Lifetime, 12.6%; Current, 1.4%; Dual, 4.9%	2744 (13–18 years old)	Significant difference	N/A	Close friends smoking (8.58), sibling smoking (3.25), teacher smoking (1.38)
Jenson et al. (2018) [23] (US)	Current, 6.4%; Dual, 3.2%	126,868 (8, 9, 11th grades)	No difference	N/A	Ethnicity (American Indian students) (3.57), sexual identity (bisexual students) (4.40), economic status (students receiving free/reduced lunch) (1.92), alcohol use (9.79), decreasing academic performance (2.47)
Kaleta et al. (2016) [57] (Poland)	Lifetime, 21.7%; Current, 27.4%	3552 (13–19 years old)	Significant difference	Higher mother’s education level (0.50), higher father’s education level (0.60), perceiving e-cigarettes as more harmful (0.30)	Father’s education level - medium (1.5), alcohol use (4.3–5.3), ever having smoked tobacco (6.7–7.5), being a current tobacco smoker (9.8–32.5), parental smoking (1.4), some friends smoking (1.4–1.5) and most friends smoking (2.3), a perception that tobacco smoking is harmful to health (1.9–3.2), perceiving e-cigarettes as less harmful (1.8–2.1)
Kinnunen et al. (2014) [58] (Finland)	Lifetime, 12.6%	3535 (12–18 years old)	Different but not statistically significant	N/A	Cigarette experimenter (8.09) and daily smoker (41.35), ever having used snus (2.96), ever having used waterpipe (2.21), vocational upper secondary school students (2.06), school performance slightly or much poorer (1.92)
Kinnunen et al. (2020) [59] (Belgium, Finland, Germany, Ireland, Italy, The Netherlands, Portugal)	Lifetime, 34%; Current, 2.7%	12,167 (14–17 years old)	Significant difference	Being older (0.77)	Parental smoking (1.28), low academic achievement (1.79), some peers smoking (2.33), most or all peers smoking (4.62)
Kintz et al. (2020) [60] (US)	Lifetime, 28.1%	2097 (11–12th grades)	No difference	N/A	Cigarette (3.46), hookah (5.85), and cigar (4.25) use
Kwon et al. (2018) [1] (US)	N/A	9853 (12–17 years old)	No difference	Perceptions of e-cigarettes as addictive (0.62) and harmful (0.40)	Internalizing problems (2.53), externalizing problems (3.47), being a rule breaker (8.43), liking frightening things (3.44), preferring unpredictable friends (4.72), having ever used alcohol (3.03) or marijuana (3.42) or other substances (1.98), household secondhand smoke exposure (1.48)
Lessard et al. (2014) [61] (US)	Lifetime, 36.9%	136 (Middle to late adolescence)	No difference	Parental monitoring (0.85)	Current cigarette use (3.88), current marijuana use (4.07), current alcohol use (7.72), peer substance use (1.34)
Maciej et al. (2012) [11] (Poland)	Lifetime, 20.9%; Current,6.9%	13.787 (15–24 years old)	Significant difference	N/A	Being male (9.0), being older (5.9), living in urban areas (8.5), ever smoked a cigarette (9.7), current cigarette smoking (15.3), parents smoking (10.0), partner smoking (15.6)
Mantey et al. (2016) [62] (US)	Lifetime, 19.8%; Current, 9.3%	22,007 (Middle and high school students)	Significant difference	Being female (0.81)	Exposure to pro e-cigarette marketing sources (1.22), being older (2.37), other tobacco use (15.66)
Mantey et al. (2019) [63] (US)	Current, 8.24%	1217 (9–12th grade)	Significant difference	N/A	Retail access to e-cigarettes (2.11–5.81)
McCabe et al. (2020) [28] (US)	Current, 11.9%	38,926 (8, 10, 12th grades)	Significant difference	N/A	Being male (1.59), average grades (1.44), binge drinking (2.46), cigarette use (4.83), marijuana use (3.08), nonmedical drug use (1.63), attending schools with a higher prevalence of smoking (1.35)
Morello et al. (2016) [64] (Argentina)	Lifetime, 7.6%	3172 (Secondary school students)	No difference	Attending a public school (0.40)	Higher sensation seeking (1.49), being a current smoker (2.58), having friends who smoke cigarettes (1.93), exposure to ads for tobacco products online (1.87)
Ofuchi et al. (2020) [65] (Thailand)	Current, 6.7%;Lifetime, 7%	6167 (13–18 years old)	Different but not statistically	N/A	Emotional abuse (1.4), physical abuse (1.4), sexual abuse (1.5), parental separation or divorce (1.36), child violence (1.8), ever having had an incarcerated household member (1.98), history of adverse childhood experience (1.5)
Park et al. (2017) [8] (Korea)	Dual, 3.5%	6307 (7–12th grades)	Significant difference	N/A	Being male (2.11), earning higher grades (3.10), higher weekly allowance (1.56), residence in urban areas (1.20), friend’s smoking (2.50), daily smoking (2.11), number of cigarettes (1.52), quitting attempts (1.52), risky drinking (1.14), lifetime drug use (1.45), lifetime sexual intercourse (1.12)
Parks et al. (2020) [66] (US)	Current, 9.99%; Dual, 5.63%	111,091 (5, 8, 9, 11th grades)	N/A	Internal assets (0.63), strong anti-smoking norms (0.88), positive teacher engagement (0.76)	Parental incarceration (0.43)
Robert Loudres et al. (2019) [29] (Malaysia)	Current, 9%	13,162 (10–19 years old)	Significant difference	N/A	Being male (4.08), age (2.64), ethnicity (2.25), cigarette smoking (13.16)
Rohde et al. (2018) [19] (US)	Lifetime, 47%	69 (14–18 years old)	Different but not statistically	Mother’s education level (0.24), addiction risk beliefs about e-cigarettes (0.46)	Combustible cigarette use (4.90)
Santistevan (2016) [67] (US)	Lifetime, 21%; Current, 4.8%	251 (13–19 years old)	N/A	N/A	Awareness of e-cigarettes through social media (15.68), shared information with peers (52.10)
Sawdey et al. (2019) [68] (US)	Current, 3.9%; Dual, 7.7%	12,460 (12–17 years old)	N/A	N/A	Low academic achievement (1.3), other tobacco use (3.7), marijuana and alcohol use (2.6), high internalizing problems (1.5), high externalizing problems (2.0), high sensation seeking (1.9), household tobacco use (1.4)
Shih et al. (2017) [69] (US)	Past year, 21.3%	2359 (High school students and college freshmen)	N/A	Neighborhood cohesion (0.83)	Neighborhood problems with alcohol and drugs (1.25), neighborhood disorganization (1.59)
Soteriades et al. (2020) [70] (Greece)	Lifetime, 12.3%; Current, 2.8%	4096 (13–15 years old)	Significant difference	N/A	Use of any combustible tobacco products (7.85), e-cigarette use by other family members (5.72), being older (2.87)
Tran (2016) [71] (US)	Lifetime, 5.6%	180 (6–9th grades)	N/A	N/A	Previous cigarette smoking experience (0.054), perception of benefits of cigarette smoking (1.14)
Trucco et al. (2021) [72] (US)	N/A	176 (14–17 years old)	N/A	N/A	Perceptions of e-cigarettes as being cool (0.28)
Unger et al. (2018) [73] (US)	Lifetime, 11.9%; Current, 2.9%	13,651 (12–17 years old)	N/A	N/A	Exposure to tobacco websites (3.0-3.2)
Veliz et al. (2017) [74] (US)	Current, 10.8%; Dual, 7.2%	4450 (12th grade)	N/A	Participation in at least one competitive sport (6.2), or three or more sports (6.4), participation in soccer (0.37)	Participation in wrestling (2.14), participation in baseball/softball (1.36)
Vogel et al. (2018) [75] (US)	N/A	173 (13–18 years old)	N/A	N/A	Percentage of friends who use e-cigarettes (0.22), past month cigarette use (0.19)
White et al. (2015) [76] (New Zealand)	Lifetime, 20%	3127 (14–15 years old)	Significant difference	N/A	Higher weekly income/allowances (2.03), current smoking (4.56), having close friends who smoke cigarettes (2.11), having used other tobacco products (2.71), having ever used marijuana (2.24), having ever engaged in binge drinking (1.87)
Wilhelm et al. (2021) [77] (US)	Current, 5.7%; Dual, 4.2%	2009 (8, 9, 11th grades)	Significant difference	Strong parental anti-smoking norms (0.19), college aspirations (0.41), internal developmental assets (0.54), parental connectedness (0.64)	Regular religious participation (2.69)
Williams et al. (2020) [78] (Canada)	Current, 22%	60,601 (14–18 years old)	Significant difference	Intramural participation among female students (0.87)	Varsity participation (1.37) for females and males (1.57), participation in both intramural and varsity sports for females (1.34) and males (1.46)
Wills et al. * (2015) [79] (US)	Lifetime, 29%; Current 18.0%	1941 (9–10th grades)	No difference	Parental support (23.3), parental monitoring (20.0), academic involvement (16.6), behavioral self-control (61.2), emotional self-control (40.4)	Parent–adolescent conflict (8.7), sensation seeking (15.8), rebelliousness (8.4), smoker prototypes (9.4), smoking expectancies (10.1), behavioral dysregulation (43.6), emotional dysregulation (24.7), peer smoking (1.5), perceiving e-cigarette as healthy (1.8), alcohol use (1.5), marijuana use (0.6), heavy drinking (0.3)
Zavala-Arciniega et al. (2019) [80] (Mexico)	Lifetime, 9.5%; Current, 10.9%	8718 (Middle school- aged students)	N/A	N/A	Being male (2.46), higher family affluence (1.13), being a regular smoker (1.81), drug use in the last year (1.89), higher technophilia (1.84), higher sensation seeking (1.31), family members using both e-cigarettes and cigarettes (1.51), being an occasional smoker (0.59)

Note 1. Numbers written inside the parentheses are either the beta coefficients or the odds ratio. * Note 2. Wills et al. (2015) did not report beta or odds ratio, but instead presented the M (SD) by groups and compare these numbers in four different user groups (e.g., Group 1 vs. Group 2). Only those variables that showed statistically significant difference in the e-cigarettes only group were included in this Table with their mean presented inside the parentheses.

## 4. Discussion

Considering these 53 studies, we estimated 15.3% as the overall pooled prevalence of international lifetime e-cigarette use, 7.7% for current use, and 4.0% for dual use among young people ranging from 9 to 25 years old. More than half of the studies reported gender differences, and males generally showed higher chances of lifetime, current, and dual e-cigarette use than females. We found the perceived cost and danger of e-cigarettes, parental monitoring, the mother’s education level, engaging in physical activity, parental support, internal developmental assets, and academic achievement as common protective factors. Moreover, we identified the following as common risk factors: friends smoking, cigarette use, alcohol use, marijuana use, family members smoking, sensation seeking, sexual intercourse, other substance/drug use, exposure to online commercials/advertisements, poor academic performance, perceiving e-cigarette as less harmful (compared to conventional cigarette use), family incarceration, and presence of retail stores nearby.

Most studies on young people’s e-cigarette use were conducted in the United States. However, there is also a growing interest in Asian countries on this issue. Aligning with this trend, Chun [88] purported that tobacco use in Western countries is decreasing, but it is rising in many Asian countries; specifically, e-cigarettes are becoming more prevalent among young people in Asia. This alarms us that the prevalence of e-cigarettes may be increasing more broadly in Asian countries. However, one must acknowledge that there are distinctive smoking cultures in Asian nations [89], and the extent of e-cigarette use, sales, and importation policies vary across Asian countries [90,91]. Therefore, researchers should design more detailed studies in the future to gain culturally sensitive insights and interpretations of e-cigarette use among youngAsian, separate from Western cultures.

The international pooled prevalence of this review (lifetime: 15.3%, current: 7.7%) is similar to the prevalence of adolescent ENDS used by Yoong et al. [17], who reported the lifetime and current ENDS use at 16.4% and 5.6%, respectively. It is important to track how this trend changes over time and be aware that 15.3% of the world’s young people have ever used an e-cigarette and that 7.7% of them are currently using e-cigarettes. This result suggests a need for active implementation of e-cigarette control and/or prevention programs for adolescents and emerging adults, separate from those preventing conventional cigarette use. For example, authorities should consider implementing a free program such as “This is Quitting,” which is proven to be an effective e-cigarette cessation text message program, especially for adolescents and young adults [92]. 

We found that current e-cigarette uses in Canada, Thailand, the United Kingdom, and the United States was higher in this review than the WHO average. However, Malaysia, Poland, South Korea, and Sweden’s prevalence were lower than the WHO average [16]. The prevalence of current e-cigarette use among young Americans in this review was consistent with Yoong et al.’s [17] systematic review (8.03% vs. 8.7%). However, we found some contrasting results concerning Canada (31.4% vs. 2.6%), the United Kingdom (13.3% vs. 1.0%), and Poland (17.1% vs. 29.9%) [17]. The methods used to estimate prevalence may account for the variances in current e-cigarette use across studies and reviews. According to the US National Institute of Mental Health [93] and Boyle [94], data collection timing, sample design, instruments, definitions, and analysis methods can change prevalence estimates. Therefore, we encourage researchers to obtain accurate estimates of e-cigarette use by choosing a representative sample from the target population, using probability sampling and validated instruments, and reporting confidence intervals [95]. If researchers do not meet any of these criteria in their studies, there is a danger of overgeneralizing results to the entire adolescent population in their countries. For instance, our study found that 96.2% of the studies did not include all age groups, but included only certain age groups (e.g., early, middle, and late adolescents). Setting a different age range for young people would make the results more difficult to interpret and may disable the comparison of the aggregated results in an international context. Thus, it is necessary to clarify and standardize the definition and criteria of young people. Further, we suggest including all age groups in the adolescent population to better represent the young population and to be able to generalize the results. In turn, we recommend that researchers present both their findings (e.g., prevalence) and the global or national prevalence to make an objective comparison of the result.

Regarding gender, approximately 70% of the studies reported gender differences and examined lifetime or current e-cigarette use between males and females. Specifically, the results found that males were more likely than females to have lifetime, current, and dual e-cigarette use. This finding is consistent with prior studies that reported significant gender differences in the prevalence and characteristics, patterns, awareness, reasons, and expectancies for e-cigarette use [96,97,98]. It further implies that e-cigarette use is a gender-specific behavior. Nonetheless, only a few studies have explored gender differences in e-cigarette use [96]. Thus, future studies should examine the role of gender in e-cigarette use to develop and implement effective prevention and intervention programs [97,98]. 

While the perceived cost and dangers of vaping were among the most recurrent protective factors in this review, the perception that cigarettes are harmful was also a risk factor. Other literature had also reported these contrasting stances [57]. For example, Amrock et al. [21] reported that 73% of adolescents believed that e-cigarettes were less harmful than cigarettes, and 47.1% thought they were less addictive than cigarettes. Perception of risks, benefits, and harm from e-cigarettes are frequently mentioned, possibly due to the underlying premise that e-cigarettes are a substitute or alternative to cigarettes. There remain conflicting assertions about whether an e-cigarette is a safer and better alternative to cigarette smoking. Some assert that e-cigarettes are effective for smoking cessation or reduction. Others purport that e-cigarettes can increase nicotine dependence and become a gateway product to other tobacco products [99]. Regardless of their effectiveness as an abstinence substitute, we cannot overlook the negative health impacts of e-cigarettes on young people [100]. Thus, we must alert the young population that e-cigarettes are not the safest alternative to smoking.

We found risk factors associated with young people’s vaping, such as peers smoking and attending a school with a high proportion of students receiving free/reduced lunch. In this regard, school-based smoking cessation programs combining social competence and social influence components [101] could be effective in preventing or reducing e-cigarette use of young people in school. Moreover, we found an association between socioeconomically disadvantaged neighborhoods and increased smoking [102], and an association among increased e-cigarette use, nearby retail stores, and communities with problems with alcohol and drugs. Hence, there should be a strict regulation of retail stores near schools and in the neighborhood area to ensure the safety of young people and to avoid them from using it. In other words, they need to be culturally responsive when designing the e-cigarette cessation programs at the neighborhood- or community-level [103]. 

In this systematic review, we found several risk factors at the societal level. These include exposure to e-cigarette commercials and marketing and an awareness of e-cigarettes through social media. This observation is consistent with previous findings that exposure to e-cigarette commercials and favorable perceptions of these commercials increased the use of e-cigarettes in adolescents [104]. E-cigarette companies actively engaging online, many young people can easily access information regarding e-cigarettes through various social media channels, which motivate them to search for vaping products without a careful consideration or validation [105,106,107]. Further, e-cigarette products are sold online with small health warning signs and a relaxed age verification process [107], which in turn increased the number of successful e-cigarette purchases by minors [108]. Currently, the restriction on sales of e-cigarettes—especially to those over a certain age—varies by country. For instance, the Tobacco & Vaping Products Act in Canada regulates the production, selling, labeling, and publicity of e-cigarettes sold in the country [109,110,111], and forbids the sale of e-cigarettes to young people under 18 years of age [112]. In addition, Canada also prohibits the advertisement of e-cigarette products that could be appealing to the youth [110]. In the US, the Prevent All Cigarette Trafficking Act and its recent amendment on Preventing Online Sales of E-Cigarettes to Children Act specifically banned the sales of cigarette products, including e-cigarettes, to minors [112,113,114]. Further, North Carolina’s age verification law prohibits the use of e-cigarettes in minor groups [115]. The Food & Drug Administration in the US has also implemented regulations on tobacco products, including e-cigarettes, and restricts the minimum age of purchase [116,117]. However, e-cigarette products are on sale without a strict age verification process in South Korea because the Tobacco Business Act does not categorize e-cigarettes as tobacco products [114,118]. It is insufficient to simply restrict the minimum purchasing age to 18 years of age; at present, age-restricted online sales protocols place the responsibility for age cross-checking on vendors and delivery companies. According to Williams et al. [112], the loosened regulation of sales of e-cigarettes online allow minors to obtain e-cigarettes more easily. The government should thus implement or make necessary changes to the current tobacco laws in a way that strictly limits the purchase and use of e-cigarettes by minors [107,108]. Kim [114] also suggested a stronger age verification process and the development of a protocol for age-restricted online sales.

While systematic reviews on e-cigarettes focus on specific outcomes, such as weight gain or its subsequent impact on cigarette smoking [119,120], reviews on prevalence and precedent factors were limited. In this regard, the significance of the current study adds to the point whereby it systematically reviewed the protective and risk factors associated with young people’s e-cigarette use in 20 different countries worldwide. Based on these results, future researchers and practitioners should have a clearer idea of what motivates and prevents these young people from using e-cigarettes. In addition, other reviews on the global prevalence of e-cigarettes were either limited to current prevalence or did not estimate dual prevalence. To overcome these limitations, this study updated the international pooled prevalence of lifetime, current, and dual e-cigarette use among young people and presented these results with a graphic representation.

However, the first limitation of the current study is concerned with the exclusion of eligible journal articles and doctoral dissertations not written in English, as well as master’s theses. If we had also analyzed these sources, the overall results of the current study (i.e., prevalence, risk factor, protective factor) could have been slightly different. Second, e-cigarette keywords used to screen the relevant studies may not have been exhaustive. For example, researchers may have used the name of specific e-cigarette products and/or brands (e.g., JUUL) in their research articles, instead of the general terms. If we had considered all these keywords during screening, the scope of this study could have been more extensive. Third, prevalence may vary across years and age groups, but this study did not consider these potential variations when pooling. However, if published year and age groups are strictly limited to certain times and/or ages, the estimated prevalence may again lead to a biased result. Fourth, we tried to estimate the international prevalence and investigate major protective and risk factors by country. However, the results are still exploratory, given that some studies have their sample population hailing from certain regions of the country. Hence, future research must validate whether this prevalence and these distinct characteristics have fully reflected the current status of each nation. Finally, future research is suggested to separately estimate the prevalence for random and non-random sampling studies and perform meta-analysis in order to strengthen the rigor of the selected studies.

## 5. Conclusions

This study estimated the international pooled prevalence of young people’s lifetime, current, and dual e-cigarette use and visualized these numbers in graphs. Based on what was found in this study, researchers would be able to address those who are more likely to use e-cigarettes and clarifies what is needed to protect adolescents from using e-cigarettes. Cigarette and e-cigarette use may share some common properties; however, there are also clear distinctions between these two products. The findings of this systematic review can be utilized as a foundational resource to enhance our understanding of e-cigarette use among young population.

## Data Availability

The data presented in this study are available in the reference list [1,4,5,8,9,11,23,28,29,30,40,41,42,43,44,45,46,47,48,49,50,51,52,53,54,55,56,57,58,59,60,61,62,63,64,65,66,67,68,69,70,71,72,73,74,75,76,77,78,79,80].

## Supplementary Materials

The following supporting information can be downloaded at: https://www.mdpi.com/article/10.3390/ijerph191811570/s1, Table S1: List of protective and risk factors associated with adolescent e-cigarette use, based on SEM.
