# Supplementary material for: An International Systematic Review of Prevalence, Risk, and Protective Factors Associated with Young People’s E-Cigarette Use
Source: Int J Environ Res Public Health. 2022 Sep 14;19(18):11570. doi: 10.3390/ijerph191811570 (PMC9517489; doi:10.3390/ijerph191811570)
Supplement: Supplementary file 1 [file ijerph-19-11570-s001.zip › ijerph-1875706-supplementary.pdf]

International Journal of
Environmental Research and Public Health

MDPI

Table S1. List of protective and risk factors associated with adolescent e-cigarette use, based on SEM.

Level		Protective factors	Risk factors
**Individual**	Status	Academic competence, , behavioral self-control, emotional self-control, positive affect, being female, internal developmental assets, being older, greater self-efficacy	Being male, being a middle school student, high level of internalizing problems, high sensation seeking, higher impulsivity, being older, higher weekly allowance, sexual identity (bisexual students), low economic status (students receiving free/reduced lunch), poor school performance, being a rule breaker, liking frightening things, higher technophilia, regular religious participation, low academic achievement, higher extraversion, history of emotional abuse, history of physical abuse, history of sexual abuse, history of child violence, history of an adverse childhood experience
	Behavior	Engaging in daily physical activity, eating a healthy diet, having never smoked, participating in at least one competitive sport or more than three sports, playing soccer	High level of externalizing problems, binge drinking, using alcohol, using cigarettes, using marijuana, using nonmedical prescription drugs, using other forms of tobacco, smoking a hookah, attempting to quit drugs, using drugs, sexual intercourse, using snus, using a water pipe, wrestling and playing baseball/softball, using cannabis, not using cannabis, using marijuana with JUUL and with other ENDS, engaging in a physical fight, liftime suicide attempt, texting or emailing while driving, being sexually active, having multiple sexual partners, drinking more soda
	Perception	Beliefs that e-cigarettes are harmful, perceptions of e-cigarettes as addictive, strong anti-smoking norms, value for conventional social norms, higher perceived costs of vaping, perceiving moderate or great risk of regularly vaping without nicotine	Perceptions of harm when using e-cigarettes, perceived less harm in using e-cigarettes, perceived less addictiveness of using e-cigarette, recognizing more cigarette brands, perception of benefits when using cigarettes, perception of e-cigarettes being cool, perception that tobacco smoking is harmful to health, awareness of e-cigarettes through social media
**Family**		Parental support, parental monitoring, mother living in the household, mother/father who never smoked tobacco, strong parental anti-smoking norms, parental connectedness, parental supervision, higher mother's education level, higher father's education level	Household tobacco use (e.g., that of father, mother, sibling), anyone living in the home using e-cigarettes (e.g., father, mother, sibling), parent–adolescent conflict, household secondhand smoke exposure, father using snuff, mother using e-cigarettes, parental incarceration, parental smoking, parental reactions (i.e., do not care about cigarette use), parental separation or divorce, having a household member who was ever incarcerated, higher family affluence
**Peer**		-	Friends smoking, number of friends who use e-cigarettes, best friends' positive reactions to e-cigarette use, having friends who have used cannabis, preferring unpredictable friends, having a best friend who smokes, peer substance use, feeling the need to smoke to fit in with peers, sharing information with peers, propensity to be easily affected by friends
**School**		Attending public school, planning to graduate from high school, positive	Attending schools with a higher prevalence of cigarette smoking, attending schools with a greater

	teacher engagement, college aspirations, intramural participation among female students, being a vocational school student	percentage of students eligible for the free and reduced lunch program, varsity participation for males, participation in both intramural and varsity sports, being a vocational upper secondary school student, teacher smoking, participating in arts vocational programs
**Community**	Not knowing anyone who uses e-cigarettes, neighborhood cohesion	Residing in urban areas, neighborhood problems with alcohol and drugs, neighborhood disorganization, presence of a retailer near schools, having seen anyone who uses e-cigarettes in the past seven days, more modelling of smoking in their social network, tobacco accessibility
**Societal**	-	Exposure to e-cigarette commercials, social norms (e.g., it's okay to use, common to use), higher exposure to online ads for tobacco products, exposure to tobacco websites, higher recall of ENDS marketing, exposure to pro e-cigarette marketing sources
